# Supplementary material for: Constructed Risk Prognosis Model Associated with Disulfidptosis lncRNAs in HCC
Source: Int J Mol Sci. 2023 Dec 18;24(24):17626. doi: 10.3390/ijms242417626 (PMC10744246; doi:10.3390/ijms242417626)
Supplement: Supplementary file 1 [file ijms-24-17626-s001.zip › ijms-2700607-supplementary.pdf]

Supplementary materials

Supplementary Table S1. Contribution of PCA variance for different subgroups.

group	Contribution of variance from principal component analysis(top100)
**All gene**	PC1(3.15%), PC2(1.73%), PC3(1.41%), PC4(1.21%), PC5(1.1%), PC6(1.03%), PC7(0.97%), PC8(0.88%), PC9(0.86%), PC10(0.79%), PC11(0.75%), PC12(0.72%), PC13(0.71%), PC14(0.68%), PC15(0.67%), PC16(0.66%), PC17(0.65%), PC18(0.61%), PC19(0.6%), PC20(0.59%), PC21(0.58%), PC22(0.58%), PC23(0.57%), PC24(0.56%), PC25(0.56%), PC26(0.55%), PC27(0.54%), PC28(0.53%), PC29(0.52%), PC30(0.52%), PC31(0.52%), PC32(0.5%), PC33(0.49%), PC34(0.49%), PC35(0.48%), PC36(0.48%), PC37(0.48%), PC38(0.46%), PC39(0.46%), PC40(0.46%), PC41(0.45%), PC42(0.45%), PC43(0.44%), PC44(0.44%), PC45(0.43%), PC46(0.43%), PC47(0.43%), PC48(0.43%), PC49(0.42%), PC50(0.41%), PC51(0.41%), PC52(0.41%), PC53(0.4%), PC54(0.4%), PC55(0.4%), PC56(0.39%), PC57(0.39%), PC58(0.39%), PC59(0.38%), PC60(0.38%), PC61(0.38%), PC62(0.38%), PC63(0.37%), PC64(0.37%), PC65(0.36%), PC66(0.36%), PC67(0.36%), PC68(0.36%), PC69(0.36%), PC70(0.35%), PC71(0.35%), PC72(0.35%), PC73(0.35%), PC74(0.34%), PC75(0.34%), PC76(0.34%), PC77(0.34%), PC78(0.34%), PC79(0.34%), PC80(0.33%), PC81(0.33%), PC82(0.33%), PC83(0.33%), PC84(0.32%), PC85(0.32%), PC86(0.32%), PC87(0.32%), PC88(0.32%), PC89(0.31%), PC90(0.31%), PC91(0.31%), PC92(0.31%), PC93(0.31%), PC94(0.31%), PC95(0.3%), PC96(0.3%), PC97(0.3%), PC98(0.3%), PC99(0.3%), PC100(0.29%).
**Disulfide gene**	PC1(19.96%),<br>PC2(13.19%),<br>PC3(12.81%),<br>PC4(10.14%),<br>PC5(9.08%),<br>PC6(9.05%),<br>PC7(8.17%),<br>PC8(7.01%),<br>PC9(6.62%),<br>PC10(4.77%).
**Disulfidptosis LncRNA**	PC1(3.59%), PC2(1.97%), PC3(1.82%), PC4(1.3%), PC5(1.21%), PC6(1.13%), PC7(1.08%), PC8(1%), PC9(0.98%), PC10(0.89%), PC11(0.84%), PC12(0.79%), PC13(0.77%), PC14(0.75%), PC15(0.74%), PC16(0.72%), PC17(0.7%), PC18(0.69%), PC19(0.68%), PC20(0.66%), PC21(0.65%), PC22(0.63%), PC23(0.62%), PC24(0.61%), PC25(0.6%), PC26(0.59%), PC27(0.58%), PC28(0.57%), PC29(0.57%), PC30(0.56%), PC31(0.55%), PC32(0.54%), PC33(0.54%), PC34(0.53%), PC35(0.53%), PC36(0.53%), PC37(0.52%), PC38(0.51%), PC39(0.5%), PC40(0.5%), PC41(0.49%), PC42(0.49%), PC43(0.48%), PC44(0.48%), PC45(0.48%), PC46(0.47%), PC47(0.46%), PC48(0.45%), PC49(0.45%), PC50(0.45%), PC51(0.44%), PC52(0.44%), PC53(0.43%), PC54(0.43%), PC55(0.43%), PC56(0.43%), PC57(0.42%), PC58(0.42%), PC59(0.41%), PC60(0.41%), PC61(0.41%), PC62(0.4%), PC63(0.4%), PC64(0.4%), PC65(0.4%), PC66(0.39%), PC67(0.39%), PC68(0.39%), PC69(0.39%), PC70(0.38%), PC71(0.38%), PC72(0.38%), PC73(0.37%), PC74(0.37%), PC75(0.37%), PC76(0.37%), PC77(0.36%), PC78(0.36%), PC79(0.36%), PC80(0.35%), PC81(0.35%), PC82(0.35%), PC83(0.35%), PC84(0.35%), PC85(0.34%), PC86(0.34%), PC87(0.34%), PC88(0.34%), PC89(0.34%), PC90(0.34%), PC91(0.33%), PC92(0.33%), PC93(0.33%), PC94(0.33%), PC95(0.32%),

	PC96(0.32%), PC97(0.32%), PC98(0.32%), PC99(0.31%), PC100(0.31%).
**Risk**	PC1(25.61%),<br>PC2(19.2%),<br>PC3(15.06%),<br>PC4(14.44%),<br>PC5(13.22%),<br>PC6(12.45%).

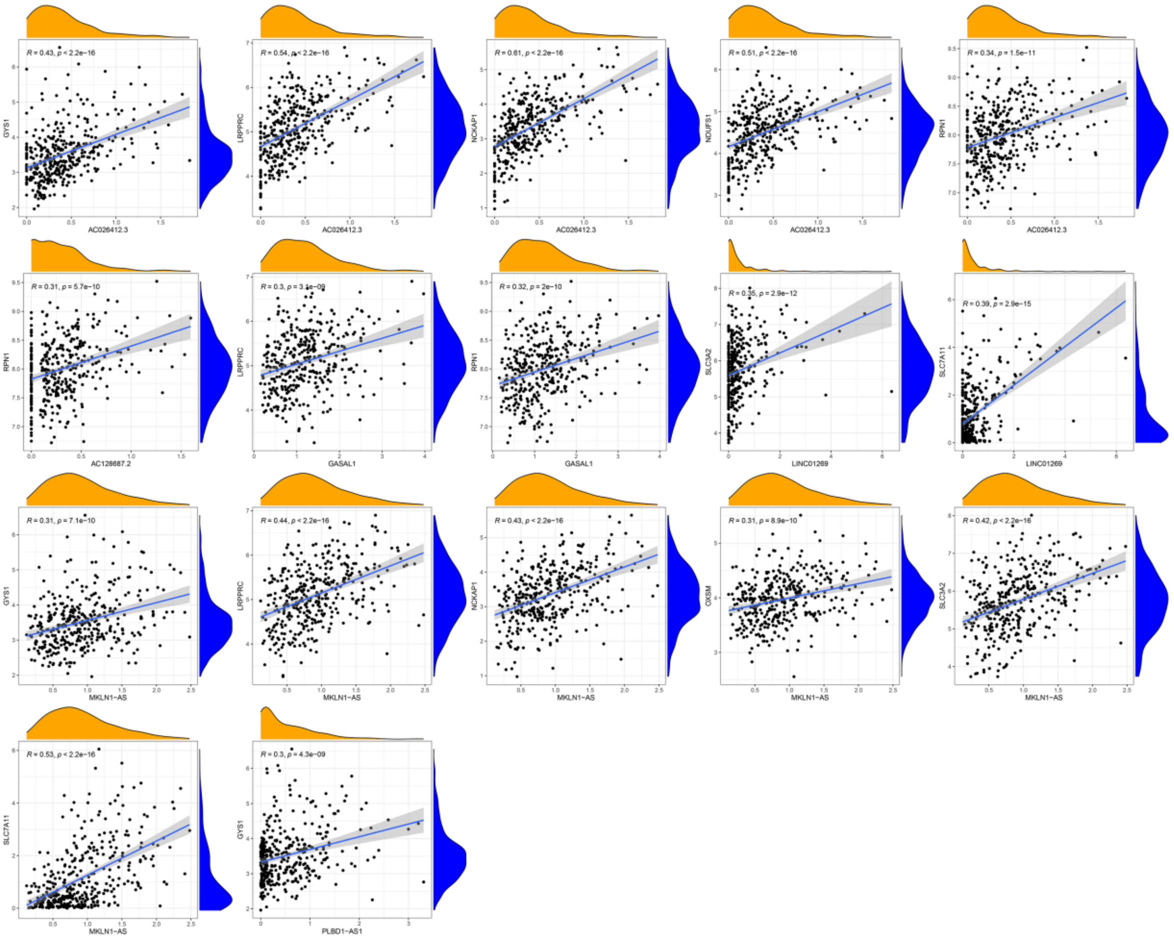

Supplementary Figure S1: Association between the expression of six lncRNAs and the expression of disulfide death genes in constructing a risk prognosis model

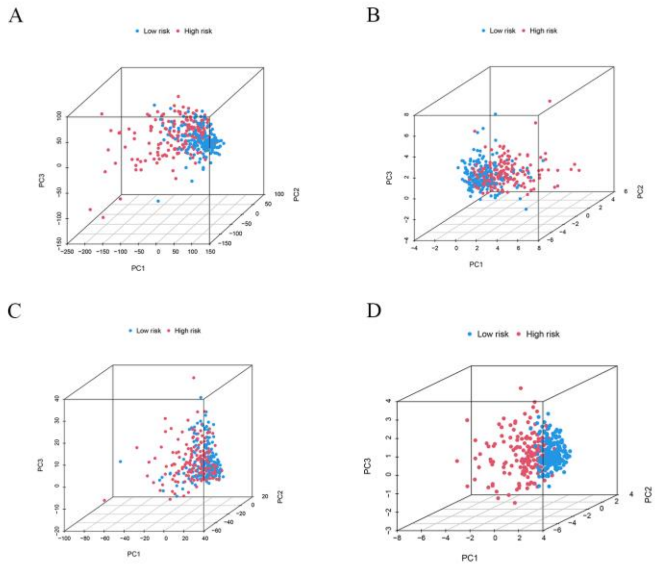

Supplementary Figure S2. The ability of PCA to assess the grouping of risk-prognostic models.

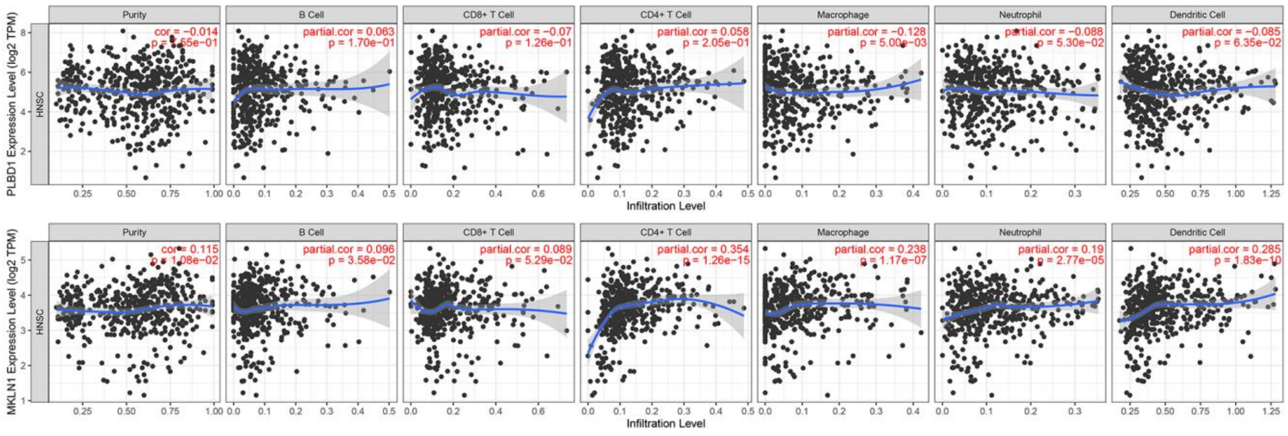

Supplementary Figure S3. Immunofunctional analysis of single lncRNAs.
